# Distinct neutrophil effector functions in response to different isolates of *Leishmania aethiopica*

**DOI:** 10.1186/s13071-024-06489-x

**Published:** 2024-11-11

**Authors:** E. Adem, E. Cruz Cervera, E. Yizengaw, Y. Takele, S. Shorter, J. A. Cotton, G. Getti, P. Kropf

**Affiliations:** 1 University of Greenwich, Medway Campus, Gillingham, UK; 2 Department of Infectious Disease, Imperial College London, London, UK; 3 Department of Medical Laboratory Science, College of Medicine and Health Science, Bahir Dar University, Bahir Dar, Ethiopia; 4 Institute of Biotechnology, Bahir Dar University, Bahir Dar, Ethiopia; 5 Amhara Public Health Institute, Bahir Dar, Ethiopia; 6 School of Biodiversity, One Health and Veterinary Medicine, College of Medical, Veterinary and Life Sciences, University of Glasgow, Glasgow, UK; 7 Present Address: Department of Comprehensive Cancer Centre, King’s College London, London, UK

**Keywords:** *Leishmania aethiopica*, Neutrophils, ROS, Phagocytosis, Apoptosis

## Abstract

**Background:**

In Ethiopia, cutaneous leishmaniasis is mainly caused by *Leishmania* (*L.*) *aethiopica* parasites and presents in three main clinical forms. It is still not clear if the host immune response plays a role in the development of these different presentations. Since neutrophils are likely to be one of the first immune cells present at the site of the sand fly bite, we set up an in vitro model of infection of neutrophils with *L. aethiopica* and assessed some of the main neutrophil effector functions: association with and internalisation of parasites, apoptosis and ROS production. We used three freshly isolated clinical isolates and one isolate that has been kept in culture for decades.

**Results:**

Our results showed by flow cytometry that all four *L. aethiopica* isolates had the ability to associate with neutrophils. The three clinical isolates of *L. aethiopica* associated more efficiently with neutrophils than the long-term cultured *L. aethiopica.* At 18 h, two distinct populations of neutrophils were identified that associated with *L. aethiopica*, CD15^high^ and CD15^low^ neutrophils. Confocal microscopy demonstrated that all isolates can be internalised. Our results also showed that all parasites induced apoptosis in *L. aethiopica*-associated neutrophils. Moreover, our results showed that after 2 h, *L. aethiopica*-associated neutrophils upregulated their production of ROS, but to a greater extent with the long-term cultured *L. aethiopica*. After 18 h of incubation, CD15^low^parasite^+^ showed an impaired ability to produce ROS compared to CD15^high^parasite^+^.

**Conclusions:**

Using this in vitro model, our results show that different *L. aethiopica* parasite isolates, most notably long-term cultured parasites, had differential effects on neutrophil effector functions.

**Graphical Abstract:**

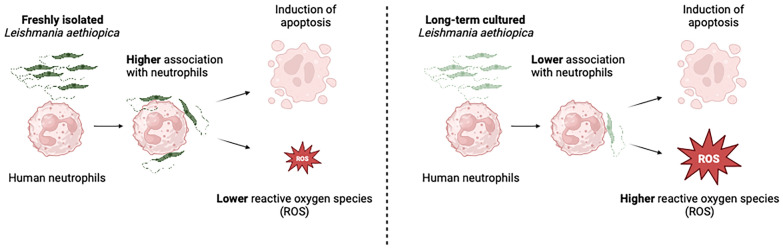

**Supplementary Information:**

The online version contains supplementary material available at 10.1186/s13071-024-06489-x.

## Background

Cutaneous leishmaniasis (CL) is caused by over 20 different species of *Leishmania* parasites, which are transmitted to their mammalian hosts during the blood meal of infected sand fly vectors. CL, the most common form of leishmaniasis, is endemic in at least 90 different countries. In 2022, over 205,000 cases were reported [1].

In Ethiopia, CL is mainly caused by *Leishmania aethiopica* [2] and presents in three main clinical forms: diffuse cutaneous leishmaniasis (DCL), characterised by numerous non-ulcerating nodules; mucocutaneous leishmaniasis (MCL), where the lesions affect the mucosa of the nose and/or mouth; and localised cutaneous leishmaniasis (LCL), characterised by small lesions that progress to ulcers. Whereas LCL usually heals spontaneously, it is not the case for DCL and MCL; both forms are difficult to treat, and relapses are frequent [3].

The mechanisms responsible for the development of these different clinical presentations of CL are not clearly understood. In a recent study, we showed that chemokine and cytokine levels in plasma as well as parasite genetic factors were not associated with different clinical presentations of CL [4]. However, only a small number of parasites isolated from DCL and MCL lesions were sequenced, which might explain why we did not identify individual genetic variants significantly associated with disease presentation. Other factors such as endosymbiont RNA viruses that are harboured by some species of *Leishmania* parasites (*Leishmania* RNA virus, LRV) can be an important virulence factor, as they cause a more severe form of CL, such as MCL [5]. LRV was also identified in five out of 11 isolates of *L. aethiopica*; the disease manifestations were not identified [6]. Host genetics has also been shown to play a role in disease development. For examples, there was an association between polymorphisms in IFN-γ and IL-4 between self-healing and chronic CL caused by *Leishmania major* [7]. Polymorphism located in the IL10 promoter was associated with increased risk of developing lesions in patients infected with *Leishmania braziliensis* [8]. However, in a recent study of 2066 CL cases caused by *L. braziliensis* and 2046 controls, genome-wide significance was not found [9]; yet, *IFNG-AS1* was of particular interest as a non-coding antisense RNA, as it has been shown to promote IFN-γ secretion in T cells and NK cells [9]. The immune response can also influence CL development [10]. In contrast to the experimental models of CL [11–13], there is no distinct T helper (Th)1 or Th2 profile in human CL. de Mesquita et al. showed higher plasma levels of cytokines such as IFNγ, IL-1β, IL-6, IL-12p70, TNFα, IL-17, IL-1RA, IL-4, IL5, IL10, IL-13, IL-2, IL-7, IL-9, and IL-15; chemokines such as eotaxin, IL-8, IP-10, MCP-1, MIP-1α, and MIP1β; and G-CSF and GM-CSF [14] in patients infected with *L. guyanensis*. In another study with *L. braziliensis*-infected patients, antigen-stimulated PBMCs produced high levels of interferon (IFN)-γ and interleukin (IL)-4 during active disease; however, after healing, the levels of IFN-γ were maintained, but those of IL-4 were low [15]. Da-Cruz et al*.* showed that antigen-specific productions of IFNγ and IL-5 by PBMCs from *L. braziliensis* patients with mucosal leishmaniasis (ML) were elevated compared to patients with CL and that no IL-4 was detectable in CL patients, but low levels were present in ML patients [16]. In DCL patients, PBMCs are unable to mount an efficient immune response [17, 18].

During any infection, neutrophils are key cells of the innate immune response that are quickly recruited following pathogen entry. They possess an array of pathogen recognition molecules, such as Toll-like receptors (TLRs), Fcγ receptor, first and third complement receptor (CR1 and CR3) and mannose receptor [19, 20]. Once engaged, neutrophils can phagocytose and kill microbes by releasing enzymes such as myeloperoxidase and elastase in the phagosome; they can degranulate and release toxic molecules, reactive oxygen species and neutrophil extracellular traps (NETs), which may kill the pathogens in the microenvironment, as well as produce cytokines and chemokines that will promote the recruitment of other immune cells and shape the adaptive immune response [21, 22].

Most of our knowledge of neutrophil effector functions during leishmaniasis is derived from mouse models. Two-photon microscopy showed that neutrophils are quickly recruited to the site of sand fly bites [23, 24]. *Leishmania amazonensis* can be killed by NETs, but other species such *L. donovani*, *L. infantum* and *L. mexicana* can survive within NETs. *Leishmania mexicana* can even multiply inside neutrophils (summarised in [25, 26]). Use of neutropenic Genista mice or depletion of neutrophils with monoclonal antibodies at the time of *Leishmania* infection showed that neutrophils can contribute to exacerbation or control of the infection, depending on several factors, such as the route of infection, genetic background of the mice and parasite strains (summarised in [25]).

In humans, it has been well documented that neutrophils are quickly recruited to the site of inflammation [27, 28]. Since the sand fly bite results in the formation of a pool of blood, neutrophils will be present at the site of infection and further attracted in numbers; they are therefore likely to be one of the first innate immune cells to interact with *Leishmania* parasites.

The interactions between neutrophils and live *L. aethiopica* parasites have not been characterised, and it is not possible to study these interactions by using a mouse model since injection of *L. aethiopica* in mice does not cause symptoms [29, 30]. Therefore, the availability of an in vitro cellular model of *L. aethiopica* infection might be useful in identifying differences in neutrophil effector functions in response to the parasites causing different clinical forms of CL.

Here, we set up an in vitro model of infection of neutrophils with *L. aethiopica* to measure some of the main effector functions of neutrophils and compare these responses between infections with freshly isolated clinical isolates of *L. aethiopica* and long-term cultured *L. aethiopica*.

## Methods

### Sample collection

Three millilitres of blood was collected in heparin tubes from healthy non-endemic controls and was processed immediately after collection: following density gradient centrifugation on Histopaque-1077 (Sigma-Aldrich, Gillingham, UK), neutrophils were isolated from the erythrocyte fraction by dextran sulphate sedimentation, as described in [31], resuspended in Roswell Park Memorial Institute Medium (RPMI) containing 5% heat-inactivated foetal bovine serum (FBS) (Sigma-Aldrich, Gillingham, UK) (complete RPMI, cRPMI), 50 IU/ml penicillin and 50 mg/ml streptomycin (Merck, Darmstadt, Germany) and immediately used for flow cytometry. Neutrophil (as defined by CD15^+^ cells) purity was > 95% (gating strategy is shown in Figure S1) and their viability, as determined by 7-aminoactinomycin D (AAD) (Biolegend, London, UK), was > 99.0% (Figure S1).

### *Leishmania* parasites

We have previously described a cohort of CL patients recruited in Nefas Mewcha, Gayint, Northern Ethiopia, from January 2019 to January 2022 [2]. Here, we used three *L. aethiopica* clinical isolates (*L. aethiopica* 1, 2 and 3) from the lesions of three different LCL patients recruited in the study described in [2]. To confirm that these isolates were *L. aethiopica*, we mapped RNA-seq reads from the isolates to the reference genome for *L. aethiopica* [32] obtained from TriTrypdb using STAR v2.7.0 [33] and then used samtools v1.17 [34] to call the consensus sequence of these mapped reads over the heat shock protein (HSP) 70 locus (LAEL147_000511500), which has been widely used to speciate *Leishmania* isolates. We compared these reconstructed sequences with previously obtained sequences from a range of *Leishmania* species (principally from [35]) using mafft v7.45 [36] to align the sequences, trimAl v2.0 [37] (with flag-strictplus), and then building a phylogeny using raxmlHPC v8.2.12 [38] (a single tree search under a GTR+gamma model of nucleotide substitution). This analysis confirmed that the isolates used here have sequences identical to *L. aethiopica* (accession FN395019) and differing by a single SNP from two other *L. aethiopica* isolates (FN395020 and FN395021). Other species formed separate groups on a phylogeny based on these sequences.

Another isolate of *L. aethiopica* (MHOM/ET/72/L100) [39, 40] was also used. Although it is not known precisely how long and how many times it had been kept frozen and in culture, it is known that it was isolated over 40 years ago [29]; this long-term cultured *L. aethiopica* isolate was therefore identified as *L. aethiopica* “laboratory” (*L. aethiopica* lab). A large stock of frozen stationary phase *L. aethiopica* 1, 2 and 3 and lab were prepared for further analysis. Once thawed, the parasites were used for a maximum of 3 weeks.

The following culture medium was used to keep the parasites in culture: M199 medium with 25 mM HEPES, 0.2 μM folic acid, 1 mM hemin, 1 mM adenine, 800 μM Biopterin, 50 IU/ml penicillin, 50 mg/ml streptomycin and 10% FBS (Sigma-Aldrich, Gillingham, UK) and the parasites were incubated at 26 °C. The parasites were passaged twice a week in new parasite medium.

Metacyclic promastigotes were isolated via agglutination with peanut agglutinin (PNA) (Merck, Darmstadt, Germany) as described previously [41] and were stained using CellTrace™ Far Red dye (FR) (Invitrogen, Loughborough, UK), using a 1 µM FR solution. Following a 20-min incubation at room temperature, the suspension was diluted 1 in 10 in cRPMI medium and incubated for a further 5 min at room temperature to quench any free dye remaining in the solution. At the end of the incubation, parasites were washed and resuspended in cRPMI medium and used for further experiments.

### Neutrophil effector functions

#### Flow cytometry

Human neutrophils (1 × 10^5^ cells/ml) were co-cultured with 1 × 10^6^ cells/ml FR labelled *L. aethiopica* isolates for 2 h, at 37 °C, 5% CO_2_, in cRPMI. Cells were then washed twice with phosphate-buffered saline (PBS, Sigma-Aldrich, Gillingham, UK), and cells to be used for the 2 h incubation were labelled with CD15^eFluor 450^ (clone MMA) (eBioscience, Santa Clara, CA, USA) for 20 min at 4 °C before being washed and immediately used for flow cytometry analysis. Cells for the 18 h incubation were resuspended in cRPMI and incubated for a further 16 h at 37 °C, 5% CO_2_, washed twice with PBS and processed as indicated above for the 2 h incubation.

#### Association of parasites with neutrophils

The % of association between neutrophils [stained with anti-human CD15^eFluor 450^ (clone MMA), eBioscience, Santa Clara, CA, USA] and FR labelled *L. aethiopica* was assessed by flow cytometry.

#### Confocal microscopy

After 2 and 18 h of incubation, cells were transferred to poly-l-lysine (0.01% solution, Sigma-Aldrich, Gillingham, UK) coated coverslips and were incubated at room temperature. After 30 min, the cells were washed twice with PBS and fixed with 2% (w/v) paraformaldehyde (Sigma-Aldrich, Gillingham, UK) for 20 min. Cells were then washed three times with PBS and incubated with CD15 (C3D-1) mouse anti-human monoclonal antibody (Thermo Fisher Scientific, Loughborough, UK) overnight at 4 °C, followed by anti-IgG (H+L) highly cross-adsorbed secondary antibody (Alexa Fluor 555) (Thermo Fisher Scientific, Loughborough, UK). After 1 h of incubation in the dark, the coverslips were washed three times with PBS and placed onto a slide containing 50 µl mounting media (VECTASHIELD mounting media, Vector Laboratories, Newark, CA, USA). Slides were visualised under a ZEISS LSM 880 confocal laser scanning microscope (Zeiss, Cambourne, UK) under 60× magnification; 1.00 Airy unit (1AU) pinhole size was used. Image acquisition was done using Zen black software and the 3D z-stack orthogonal images were analysed by Zen 3.3 (blue edition) software.

#### Apoptosis

The PE-Annexin V/7-amino-actinomycin D (7-AAD) apoptosis detection kit (BioLegend, Greenwood, UK) was used to detect apoptosis according to the manufacturer’s protocol. Briefly, following the 2 and 18 h incubations with the parasites and labelling of neutrophils with CD15 antibody as described above, cells were washed and re-suspended with 100 µl Annexin V binding buffer, and 5 µl PE-Annexin V and 5 µl 7AAD (7-amino-actinomycin D) were added to the cell suspension. After 15 min of incubation in the dark at room temperature, a further 400 µl Annexin V binding buffer was added and the cells were immediately analysed by flow cytometry.

#### ROS detection assay

ROS-ID™ Total ROS detection kit (Enzo Life Sciences, Farmingdale, NY, USA) was used to evaluate the production of ROS by neutrophils according to the manufacturer’s protocol. Briefly, following the 2 and 18 h incubations with the parasites and labelling of neutrophils with CD15 antibody as described above, cells were washed; 25 nM ROS detection solution in 500 µl PBS was added to the cells, which were incubated for 30 min at 37 °C, 5% CO_2_, and immediately used for flow cytometry analysis.

Flow cytometry acquisition was performed using an LSRII (BD Biosciences, Wokingham, UK) and data were analysed using Summit v4.3 software (Beckman Coulter, Brea, CA, USA).

### Statistical analysis

Data were evaluated for statistical differences as specified in the legend of each figure, using GraphPad Prism 10 (San Diego, CA, USA). The following tests were used: Mann-Whitney and Kruskal-Wallis. Results are expressed as mean ± SD. Differences were considered statistically significant at *P* < 0.05. *P < 0.05, **P < 0.01, ***P < 0.001 and ****P < 0.0001.

## Results

### Association of *aethiopica* parasites with neutrophils

**Fig. 1:**
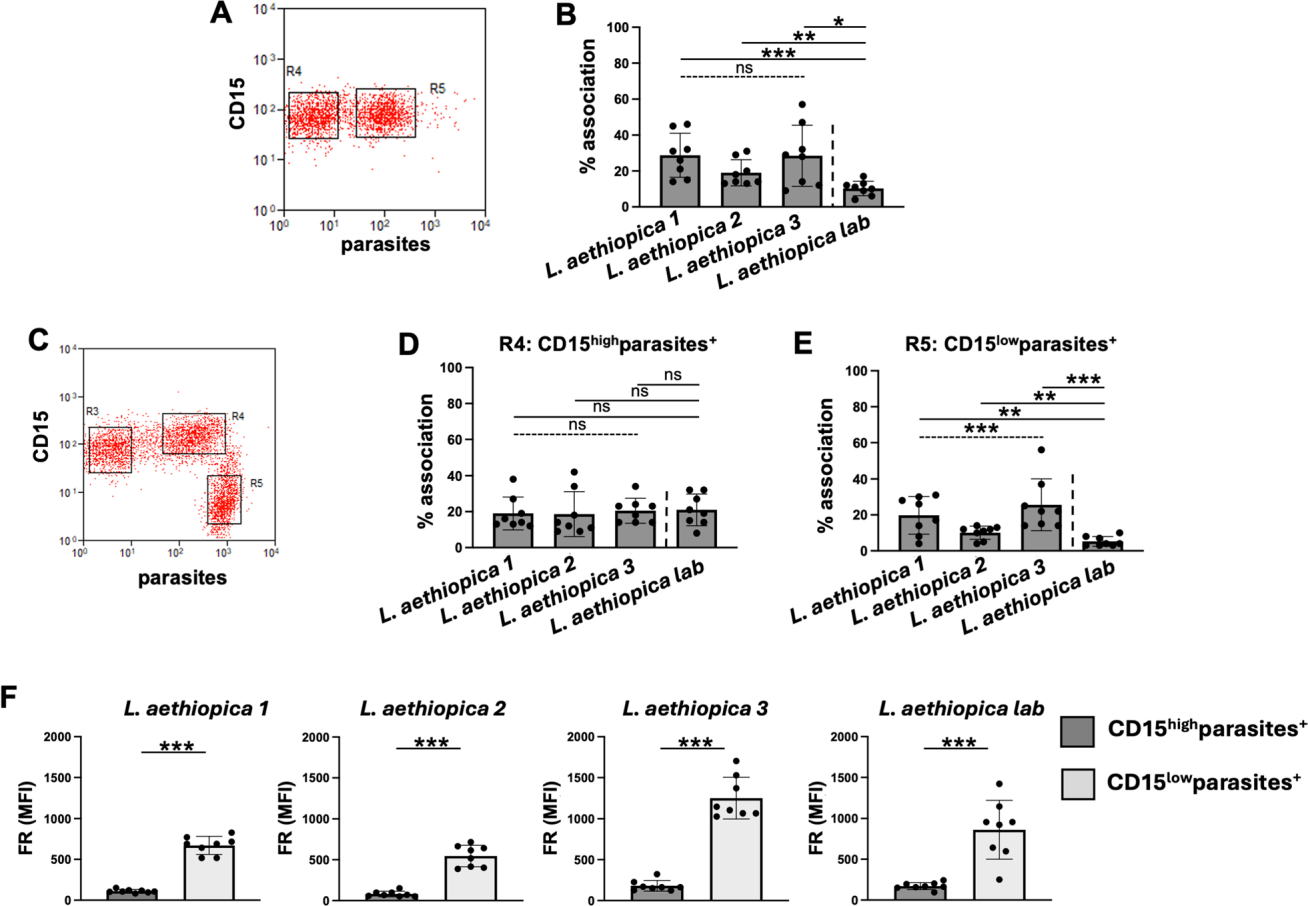
Association of neutrophils with different isolates of *Leishmania aethiopica*; 1 × 10^5^ cells/ml neutrophils were co-cultured with 1 × 10^6^ cells/ml FR labelled *L. aethiopica* isolates for 2 h (**A**, **B**) and 18 h (**C**–**F**). The percentages of neutrophils associated with *L. aethiopica* were determined by flow cytometry. **A** Dot plot showing neutrophils unassociated (gate R4) and associated (gate R5) with *L. aethiopica*. **B** % of neutrophils associated with the four different isolates of *L. aethiopica*. **C** Dot plot showing the three different population of neutrophils: CD15^intermediate (int)^parasite^−^ (gate R3), CD15^high^parasite^+^ (gate R4) and CD15^low^parasite^+^ (gate R5). **D** % of CD15^high^parasite^+^ associated with the four different isolates of *L. aethiopica.* **E** % of CD15^low^parasite^+^ associated with the four different isolates of *L. aethiopica.* **F** Comparison in FR MFI between CD15^high^parasite^+^ and CD15^low^parasite^+^ for each *L. aethiopica*. Data are presented as scatter plot with bar (mean with standard deviation), with each dot representing the value for one experiment. Statistical differences were determined using Kruskal-Wallis (dotted line) and Mann-Whitney (solid line) tests

We first assessed by flow cytometry whether the three clinical and laboratory isolates of *L. aethiopica* can associate with neutrophils after 2 h of co-incubation; we chose a 2 h incubation as after 2 h the percentages of association between neutrophils and parasites were plateauing. Results presented in Fig. 1A, B show that *L. aethiopica* can associate with neutrophils and that the percentages of the *L. aethiopica* lab associated with neutrophils were significantly lower than for the three clinical isolates (Fig. 1B and Table 1). There were no significant differences among the three clinical isolates (Fig. 1B and Table 1).

**Table 1:**
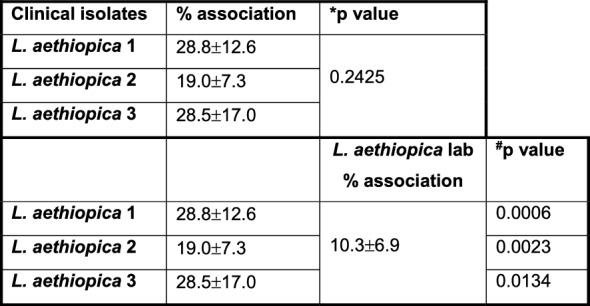
Association of neutrophils with *Leishmania aethiopica* following 2 h of co-incubation

1 × 10^5^ cells/ml neutrophils were co-cultured with 1 × 10^6^ cells/ml FR labelled *L. aethiopica* isolates for 2 h. The percentages of neutrophils associated with *L. aethiopica* were determined by flow cytometry. Statistical differences were determined using Kruskal-Wallis (*) and Mann-Whitney (^#^) tests

Depending on culture conditions, such as low levels of glucose or oxygen, neutrophils can have a short life span in vitro [42, 43]. However, for co-culture of neutrophils with *Leishmania* parasites, it has been shown to delays apoptosis for up to 24 h [44], suggesting that it prolongs neutrophil survival. Here, we first assessed whether their ability to associate with parasites was maintained after 18 h of co-culture. As shown in Fig. 1C, there were two distinct populations of neutrophils associated with the parasites: CD15^high^ (gate R4) and CD15^low^ (gate R5). This was the case for all four isolates of *L. aethiopica* (data not shown). There were no significant differences between different isolates in the percentages of parasites associated with neutrophils in the CD15^high^parasites+ (Fig. 1D and Table 2). However, the percentages of CD15^low^ cells associated with *L. aethiopica* 2 were significantly lower compared to *L. aethiopica* 3 (Fig. 1E and Table 3). Furthermore, the percentages of the three clinical isolates associated with CD15^low^ cells were all significantly higher compared to *L. aethiopica* lab (Fig. 1E and Table 3). Since there were differences in FR intensities among the four parasite isolates (Figure S2), it was not possible to compare the intensity of association between the neutrophils and different isolates. However, it was possible to compare the CD15^high^ and the CD15^low^ populations for each parasite isolate. As shown in Fig. 1F, the median fluorescent intensity (MFI) of the FR labelled parasites were always significantly higher in the CD15^low^ population, suggesting that more parasites were associated with the CD15^low^ population.

**Table 2:**
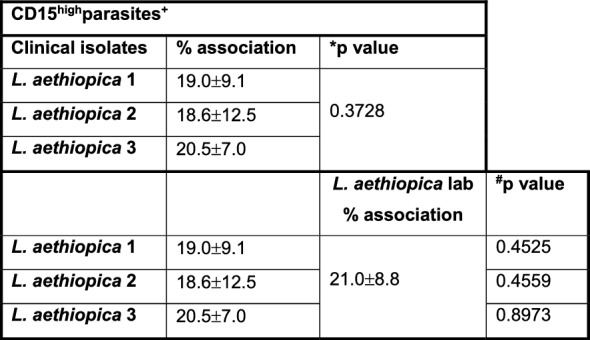
Comparison of the association of CD15^high^ neutrophils with the different *Leishmania aethiopica* isolates after 18 h

1 × 10^5^ cells/ml neutrophils were co-cultured with 1 × 10^6^ cells/ml FR labelled *L. aethiopica* isolates for 18 h. The percentages of CD15^high^ neutrophils associated with *L. aethiopica* were determined by flow cytometry. Statistical differences were determined using Kruskal-Wallis (*) and Mann-Whitney (^#^) tests

**Table 3:**
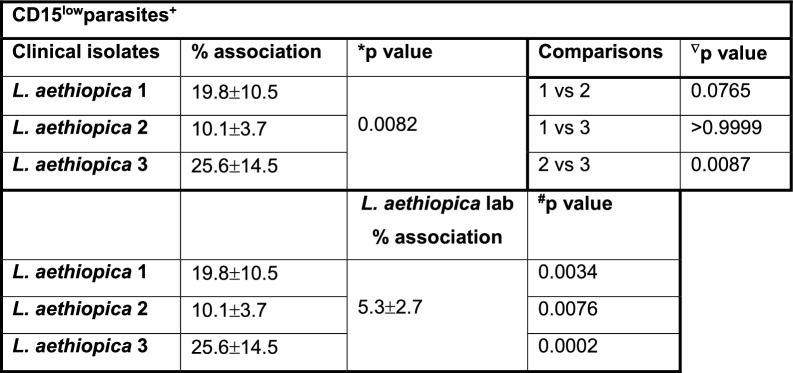
Comparison of the association of CD15^low^ neutrophils with the different *Leishmania aethiopica* isolates after 18 h

1 × 10^5^ cells/ml neutrophils were co-cultured with 1 × 10^6^ cells/ml FR labelled *L. aethiopica* isolates for 18 h. The percentages of neutrophils associated with *L. aethiopica* were determined by flow cytometry. Statistical differences were determined using Kruskal–Wallis (*), Dunn’s multiple comparison (^∇^) and Mann–Whitney (^#^) tests

### Internalisation of *L. aethiopica* by neutrophils

**Fig. 2:**
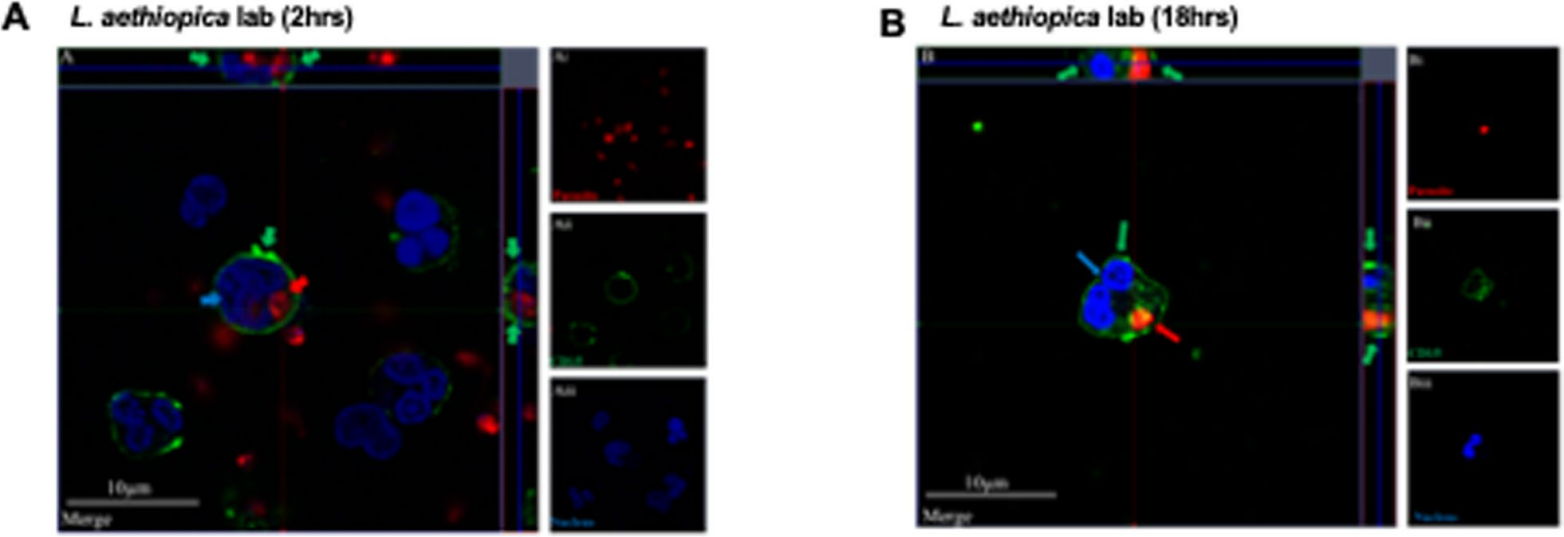
Internalisation of *Leishmania aethiopica* by neutrophils; 1 × 10^5^ cells/ml neutrophils were co-cultured with 1 × 10^6^ cells/ml FR labelled *L. aethiopica* lab for 2 h (**A**) and 18 h (**B**) and cells were labelled as described in “Methods”. The red arrows point to the parasite (FR), the green arrows to the CD15 (Alexa Fluor 555) and the blue arrow to the nucleus (DAPI). These are representative images of at least three independent experiments

Next, confocal microscopy was used to demonstrate that *L. aethiopica* is internalised by neutrophils and does not only associate, as shown by flow cytometry. Results presented in Fig. 2 (*L. aethiopica* lab) and Figures S3 (*L. aethiopica* 1, 2 and 3) show that *L. aethiopica* parasites were internalised within neutrophils following 2 and 18 h of incubation. The top and right panels, both delineated by a grey line, show the horizontal and vertical view of the z-stack. Both show that the internalised parasites are surrounded by CD15^+^ neutrophil membrane.

### Neutrophil apoptosis

To determine how co-culture of neutrophils with *L. aethiopica* impacts their ability to undergo apoptosis, we measured the percentages of apoptotic cells [as defined by Annexin V^+^ 7-AAD^−^ (early apoptosis) or Annexin V^+^ 7-AAD^+^ (late apoptosis) neutrophils]. The gating strategy is shown in Figure S4. As shown in Figure S4D, E, most gated apoptotic neutrophils were in the early stage of apoptosis. Following 2 h of incubation, the percentages of apoptotic cells were systematically increased in neutrophils associated or not with all *L. aethiopica* isolates compared to neutrophils alone (Table S1). Of note, the % of apoptotic neutrophils were significantly higher in neutrophils associated with the three clinical isolates compared to unassociated neutrophils; this was however not the case with *L. aethiopica* lab (Table S2).

**Fig. 3:**
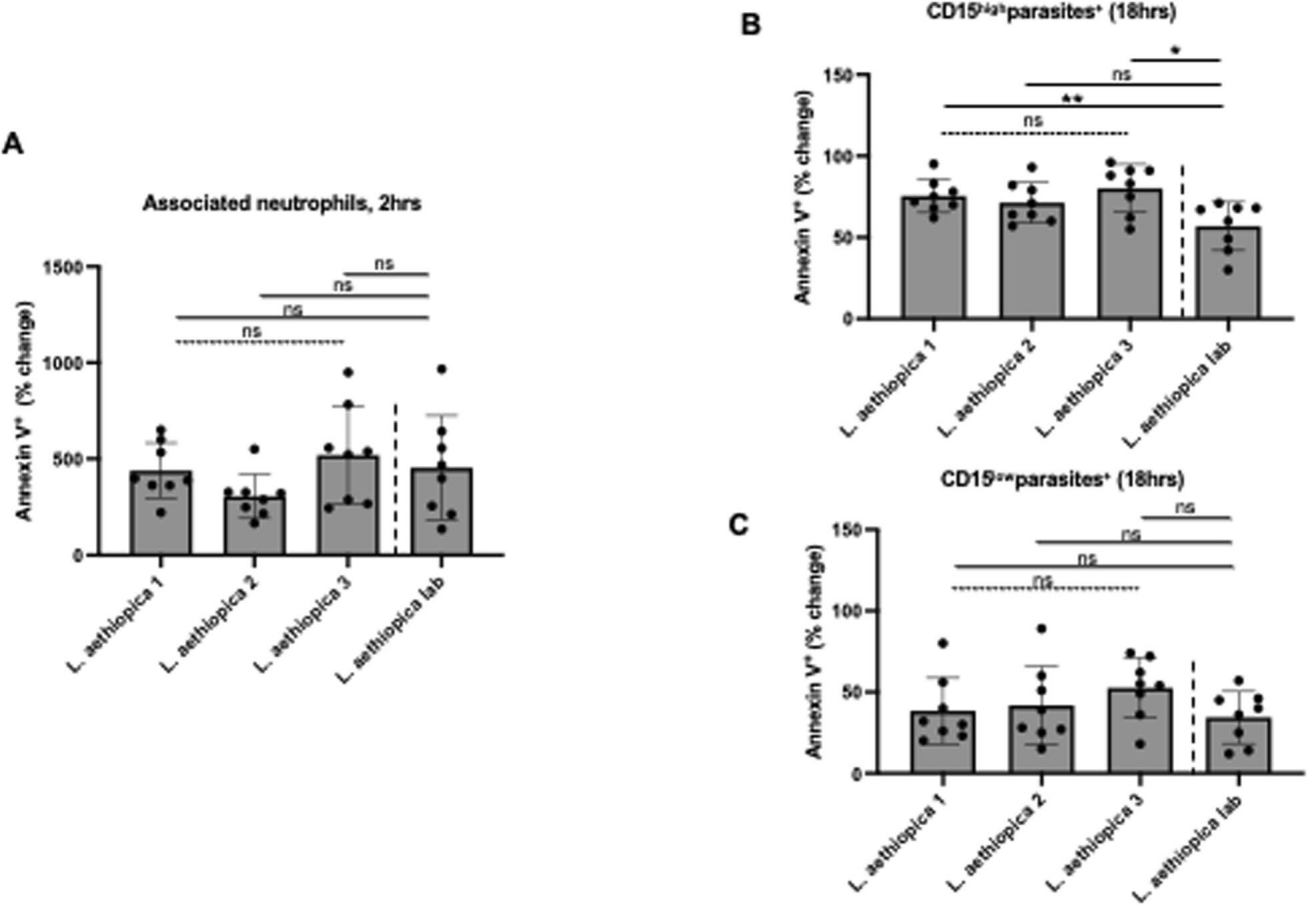
% change in apoptotic neutrophils between the different parasite isolates; 1 × 10^5^ cells/ml neutrophils were co-cultured with 1 × 10^6^ cells/ml FR labelled *L. aethiopica* isolates for 2 h (**A**) and 18 h (**B**, **C**). The % change was measured by deducting the % apoptotic (as defined by Annexin V^+^7-AAD^−^) CD15^high^ neutrophils co-cultured with the parasites from the % of apoptotic neutrophils cultured in the absence of parasites. Data are presented as scatter plot with bar (mean with standard deviation), with each dot representing the value for one experiment. Statistical differences were determined using Kruskal-Wallis (dotted line) and Mann-Whitney (solid line) tests

To be able to compare the % of neutrophils undergoing apoptosis following incubation with the different parasite isolates, % changes in apoptosis were assessed between baseline (neutrophils incubated without parasites) and those neutrophils co-incubated with *L. aethiopica*. Results show that after 2 h of incubation, there was no significant difference between any isolates in % change in apoptosis by neutrophils associated (Fig. 3A) or not (data not shown) with *L. aethiopica* between all isolates.

After 18 h of incubation (gating strategy shown in Figure S5), most gated apoptotic neutrophils were in the early stage of apoptosis (Figure S5). Results presented in Table S3 show that for all isolates, the % Annexin V^+^ 7-AAD^−^ neutrophils were similar between all four parasite isolates in CD15^int^parasites^−^ and lower in CD15^high^parasites^+^ and CD15^low^parasites^+^ compared to baseline. When comparing the % Annexin V^+^ 7-AAD^−^ neutrophils among the three populations of neutrophils, it was always highest in the CD15^int^parasites^+^ and lowest in the CD15^low^parasites^+^ (Table S4).

There were no significant differences in % change in the frequency of apoptotic cells among the four different isolates in CD15^int^parasite^−^ (data not shown). In the CD15^high^parasites^+^, the % change was significantly higher with *L. aethiopica* 1 and 3, but not 2, compared to *L. aethiopica* lab (Fig. 3B and Table 4). However, there were no significant differences in % change between any parasites isolates in the CD15^low^parasites^+^ (Fig. 3C).

**Table 4:**
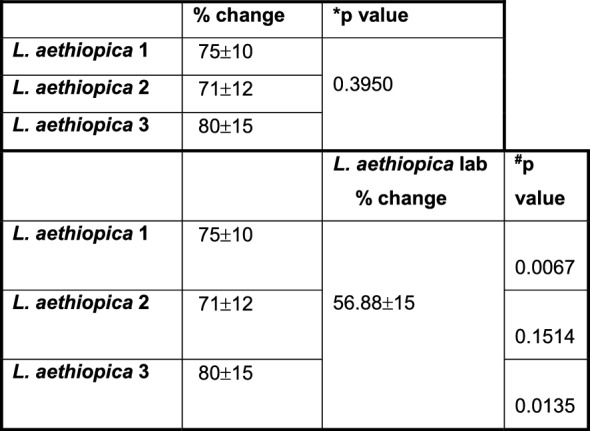
% change in apoptotic CD15^high^ neutrophils between the different parasite isolates after 18 h

1 × 10^5^ cells/ml neutrophils were cultured in the presence or the absence of 1 × 10^6^ cells/ml FR labelled *L. aethiopica* isolates for 18 h. The % change was measured by deducting the % apoptotic (as defined by Annexin V^+^7AAD^−^) CD15^high^ neutrophils co-cultured with the parasites from the % of apoptotic neutrophils cultured in the absence of parasites. Statistical differences were determined using Kruskal-Wallis (*) and Mann-Whitney (^#^) tests

### ROS production by *L. aethiopica*-associated neutrophils

Next, we assessed the ability of neutrophils to upregulate ROS following co-incubation with *L. aethiopica* (gating strategy shown in Figure S6). Following 2 h of incubation, ROS production (MFI) was systematically significantly increased in *L. aethiopica*-associated neutrophils, but it was not significantly higher in unassociated neutrophils, except for a borderline difference with *L. aethiopica* 2 (*P* = 0.0433, Table S5). Of note, the levels of ROS production (MFI) were significantly higher in *L. aethiopica*-associated neutrophils than in unassociated ones (Table S6).

**Fig. 4:**
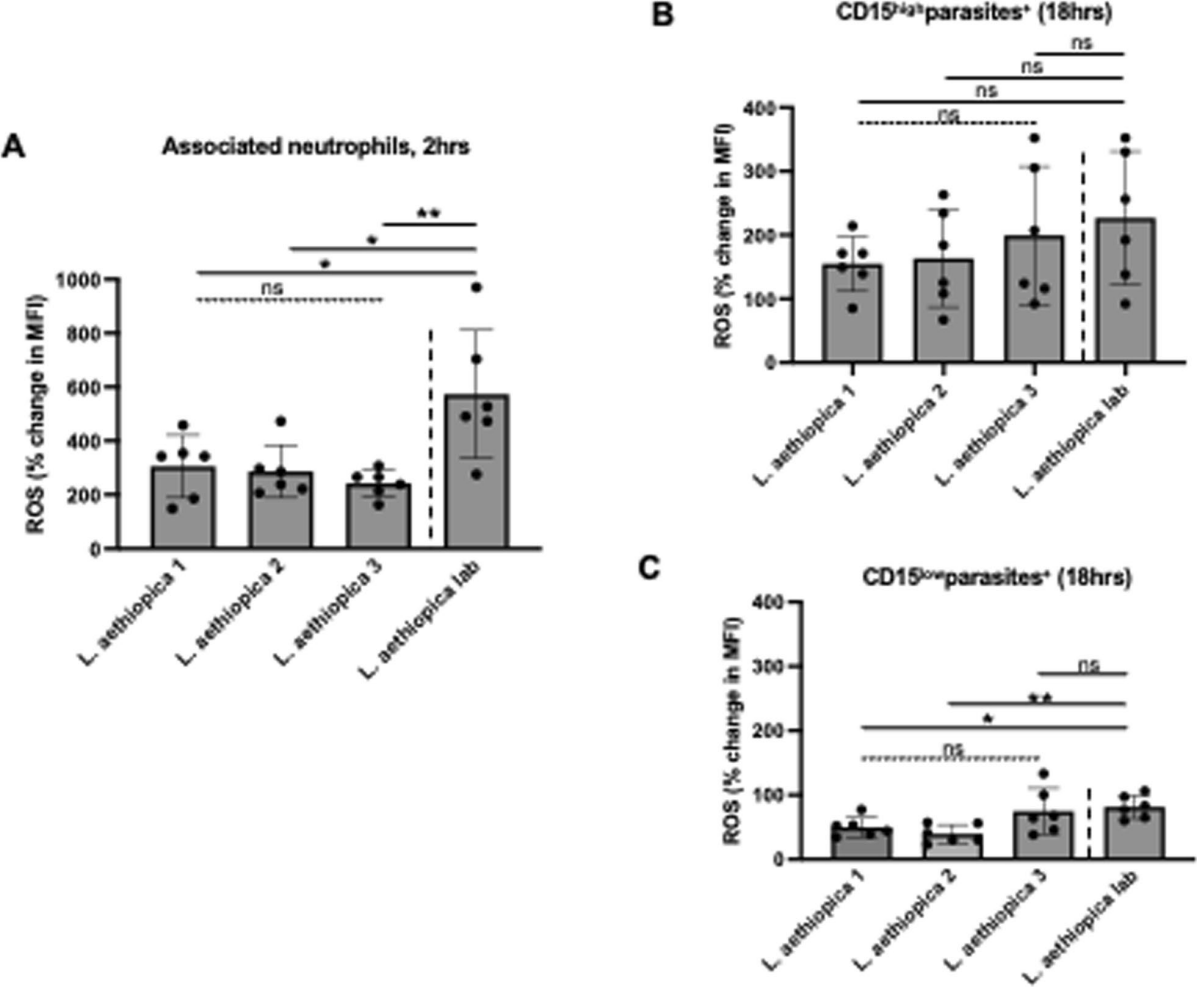
% change in ROS MFI in neutrophils between the different parasite isolates; 1 × 10^5^ cells/ml neutrophils were co-cultured with 1 × 10^6^ cells/ml FR labelled *L. aethiopica* isolates for 2 h (**A**) and 18 h (**B**, **C**). The % change was measured by deducting the ROS MFI in the neutrophils co-cultured with the parasites from the ROS MFI in the neutrophils cultured in the absence of parasites. Data are presented as scatter plot with bar (mean with standard deviation), with each dot representing the value for one experiment. Statistical differences were determined using Kruskal-Wallis (dotted line) and Mann-Whitney (solid line) tests

To be able to compare the levels of ROS production by neutrophils between the different parasite isolates, % changes in ROS production were assessed between baseline (neutrophils incubated without parasites) and those neutrophils co-incubated with *L. aethiopica*. Results shown in Fig. 4A and Table 5 show that after 2 h of incubation, there was no significant difference in % change in ROS production by neutrophils associated with *L. aethiopica* among the three clinical isolates. However, it was significantly higher for *L. aethiopica* lab compared to the clinical isolates (Fig. 4A and Table 5). There was no significant difference in ROS production among all four isolates in the *L. aethiopica*-unassociated neutrophils (data not shown).

**Table 5:**
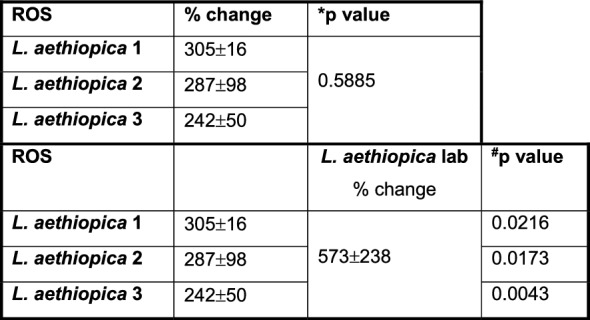
% change in ROS MFI in neutrophils between the different parasite isolates after 2 h

1 × 10^5^ cells/ml neutrophils were cultured in the presence or the absence of 1 × 10^6^ cells/ml FR labelled *L. aethiopica* isolates for 2 h. The % change was measured by deducting the ROS MFI in the neutrophils co-cultured with the parasites from the ROS MFI in the neutrophils cultured in the absence of parasites. Statistical differences were determined using Kruskal-Wallis (*) and Mann-Whitney (^#^) tests

After 18 h of incubation (gating strategy shown in Figure S7), results presented in Table S7 show that ROS MFI was similar when comparing baseline with CD15^int^parasites^−^ and CD15^high^parasites^+^ neutrophils for all four *L. aethiopica* isolates. However, ROS MFI was significantly lower in CD15^low^parasites^+^ compared to baseline for all three clinical isolates, but not *L. aethiopica* lab (Table S7). When comparing ROS production between the three populations of neutrophils, the CD15^high^parasites^+^ always produced the highest levels of ROS compared to CD15^low^parasites^+^ (Table S8). There were no significant differences in % change among the four different isolates in CD15^high^parasite^+^ (Fig. 4B) and CD15^int^parasite^−^ (data not shown). However, the % changes in ROS production in CD15^low^parasite^+^ neutrophils were lower with *L. aethiopica* 1 and 2, but not 3, compared to *L. aethiopica* lab (Fig. 4C and Table 6).

**Table 6:**
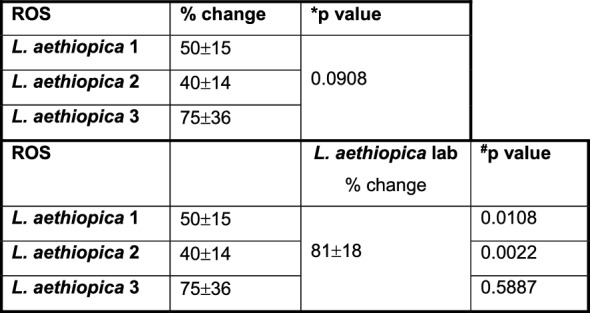
% change in ROS MFI in CD15^low^ neutrophils between the different parasite isolates after 18 h

1 × 10^5^ cells/ml neutrophils were cultured in the presence or the absence of 1 × 10^6^ cells/ml FR labelled *L. aethiopica* isolates for 18 h. The % change was measured by deducting the ROS MFI in the CD15^low^ neutrophils co-cultured with the parasites from the ROS MFI in the CD15^low^ neutrophils cultured in the absence of parasites. Statistical differences were determined using Kruskal-Wallis (*) and Mann-Whitney (^#^) tests

## Discussion

Here we set up an in vitro model of infection of human neutrophils with *L. aethiopica* and show that parasites can be phagocytosed by neutrophils and that association of neutrophils with *L. aethiopica* modulates apoptosis and ROS production. Our results also show that *L. aethiopica* lab associated less and induced more ROS production compared to freshly isolated *L. aethiopica*. We also identify some differences in the percentages of associated parasites among the three clinical isolates. Many studies imply that association as shown by flow cytometry equates to phagocytosis. However, by using flow cytometry alone, it is not possible to differentiate cells that are bound to neutrophils from those that have been internalised [45]. Techniques such as imaging flow cytometry or confocal microscopy need to be used to validate internalisation [45].

Little is known about the mechanisms influencing the different clinical presentations of cutaneous lesions caused by *L. aethiopica*. It has been previously suggested that differences in parasites are associated with the different clinical manifestations [46–48]. A later study suggested that the genetic variability did not correlate with the different manifestations [49]. In agreement with these results, in the largest study to date analysing genetic variations between *L. aethiopica* isolated from different lesions, we found no individual genetic variants were significantly associated with disease presentation [4]. It is also possible that *Leishmania* RNA viruses play a role in the development of disease manifestations [5, 6, 50].

Here, we used three *L. aethiopica* parasites freshly isolated from patients with localised cutaneous leishmaniasis (LCL) and one isolate that had been kept in culture for decades; the history of the number of in vitro passages and its origin have been poorly characterised [29]. *Leishmania aethiopica* parasites cannot be passaged in vivo [29, 30] to maintain virulence [51–53]. Therefore, to minimise variations due to the time in culture, once the parasites were growing from the cultured skin scrapings from CL patients, they were immediately frozen and shipped to the UK. Large stocks of parasites were produced and frozen, and once thawed, the parasites were kept in culture for a maximum of 3 weeks before a new tube was thawed. It has been shown that the metacyclogenesis of parasites is key in determining their virulence [54]. Therefore, to maximise our control over the stage of parasites used in these experiments, parasites were grown to a stationary phase and metacyclic parasites were purified using PNA.

Long-term in vitro culture has been associated with the loss of virulence of *Leishmania* parasites in both phagocytic cells, as shown by fewer amastigotes per cells [51–53], and animal models, as shown by a lower parasite burden or smaller lesions [53, 55, 56]. Several virulence factors have been identified in *Leishmania* parasites [57]. In particular, reduced expressions of lipophosphoglycan (LPG) and glycoprotein (GP) 63 have been shown to result from long-term in vitro culture [58, 59]. Both these molecules are important in the phagocytosis of *Leishmania* parasites [19, 60]. This might therefore explain why *L. aethiopica* lab associated significantly less with neutrophils than with the freshly isolated *L. aethiopica* parasites.

Interestingly, following 18 h of incubation, in addition to a population of neutrophils that did not associate with *L. aethiopica*, two other populations of neutrophils associated with *L. aethiopica* were identified based on the expression levels of CD15. These results show that following co-incubation of neutrophils with *L. aethiopica*, at least three populations of neutrophils can be identified that have different abilities to associate with the parasites. The heterogeneity of neutrophils is now well recognised [22, 61, 62]. scRNAseq analysis of circulating neutrophils showed three major populations [63]. Another study showed a high level of heterogeneity of neutrophils following phenotypic characterisation of peripheral neutrophils in healthy individuals and compared to patients with different pathological conditions [64]. We also found differences in the percentages of associated CD15^low^ neutrophils among the three clinical isolates at 18 h; these results suggest that even though all three clinical isolates were obtained from lesions of LCL patients, there still might be differences between these parasites.

Infection of neutrophils by *L. major* and *L. infantum* has been shown to result in delayed apoptosis over time, suggesting that the parasites prolong the survival of neutrophils, thereby allowing the intracellular parasites to survive longer [44, 65]. Our results show that after 2 h of incubation, there were increased percentages of apoptotic associated and unassociated neutrophils compared to baseline. This was in contrast to 18 h, when there were significantly lower percentages of apoptotic associated neutrophils (CD15^high^ and CD15^low^), but not unassociated neutrophils (CD15^int^), compared to baseline. The study by van Zandbergen et al. showed that apoptotic neutrophils can be phagocytosed by macrophages and that the phagocytosed parasites are then able to survive and multiply in these macrophages [66]. It is therefore possible that it will also be the case with *L. aethiopica* and that apoptotic neutrophils will be taken up by phagocytic cells, monocytes in the blood and macrophages if they enter tissues. It has been previously shown that compared to uninfected neutrophils, *L*. *major*-infected neutrophils have an increased ability to take up noninfected apoptotic cells [67]; this was associated with the downregulation of ROS production and better survival of parasites in the neutrophils. Thus, the high percentages of unassociated neutrophils identified in our study might contribute to better survival of the intracellular parasites. Whereas it has been shown that *Leishmania*-infected neutrophils undergo apoptosis [44, 65–67], there is little information about unassociated neutrophils undergoing apoptosis. They might become apoptotic because of a transient contact with *Leishmania* parasites, induction of ROS or production of cytokines such as TNFα (summarised in [68]).

In our study, after 18 h, there were fewer apoptotic cells in associated neutrophils compared to baseline. This contradicts the study by Oualha et al., as they show an increase in the percentages of apoptotic neutrophils following co-incubation with *L. major* and *L. infantum* compared to baseline. This might be due to differences in the experimental conditions, such as different parasite strains, a tenfold higher number of neutrophils co-cultured with parasites and differences in the stage of the parasites, as in this study, they did not use PNA to isolate the metacyclic parasites [65].

It has been previously shown that infection of neutrophils with different parasite species such as *L. infantum* [69, 70], *L. braziliensis* [71] and *L. donovani* [72] results in upregulation of ROS. One study showed higher levels of ROS by neutrophils in response to *L. aethiopica*; however, lysate and not live *L. aethiopica* were used in this study [73]. In our study, ROS was upregulated in neutrophils following 2 h of co-culture with all parasite isolates. ROS was also increased in unassociated neutrophils; this is likely caused by transient contact with the parasites during the 2 h co-culture. In contrast, after 18 h, ROS was similar to baseline levels in CD15^int^parasites^−^ and in CD15^high^parasites^+^ neutrophils and significantly lower in CD15^low^parasites^+^. The latter subpopulation was also the population that had the highest % of associated parasites, suggesting that the parasites might manipulate the neutrophils to limit the levels of ROS production to survive more efficiently. In support of these data, ROS had already been shown to be reduced following exposure of *L. major*-infected neutrophils to apoptotic cells [67]. Furthermore, a study by Mollinedo et al. has shown that *L. major*- and *L. donovani*-containing phagosomes do not fuse with specific and tertiary granules, thereby preventing the production of ROS [74]. Al Tuwaijri et al. have also shown that different preparations of *L. major* parasites reduced the respiratory burst by neutrophils [75].

Of note, *L. aethiopica* lab induced higher levels of ROS at both 2 and 18 h compared to the clinical isolates. Both LPG [76] and GP63 [77] have been shown to inhibit the oxidative burst. As discussed above, the extensive time in culture might have resulted in lower expression levels of LPG and GP63 on *L. aethiopica* lab and thereby impacted the levels of ROS production.

## Conclusions

In this study we characterised, for the first time to our knowledge, neutrophil effector functions in response to different isolates of *L. aethiopica*. Our results show that care should be taken when using parasites that have been kept in culture for a long time and highlight that a more standardised way to isolate primary cells and infectious metacyclic parasites should be used.

In the absence of a mouse model, in vitro infection of phagocytic cells might identify different functional profiles that could shed light on the different presentation of lesions caused by *L. aethiopica*.

## Supplementary Information

Supplementary Material 1.

Supplementary Material 2.

Supplementary Material 3.

Supplementary Material 4.

Supplementary Material 5.

Supplementary Material 6.

Supplementary Material 7.

Supplementary Material 8.

## Data Availability

The datasets supporting the conclusions of this article are included within the article and its additional files.
