# Supplementary material for: Distinct neutrophil effector functions in response to different isolates of Leishmania aethiopica
Source: Parasit Vectors. 2024 Nov 11;17:461. doi: 10.1186/s13071-024-06489-x (PMC11555981; doi:10.1186/s13071-024-06489-x)
Supplement: Supplementary file 1 — Supplementary Material 1. [file 13071_2024_6489_MOESM1_ESM.docx]

**Table S1: % of apoptotic neutrophils following 2 hrs of incubation**

| **% Annexin V^+^ 7AAD^-^ neutrophils** | **Baseline**  **(%)** | **Associated**  **(%)** | **^#^p value** |
| --- | --- | --- | --- |
| ***L. aethiopica* 1** | 7.9±2.2 | 32.8±8.8 | 0.0002 |
| ***L. aethiopica* 2** |  | 23.0±7.5 | 0.0002 |
| ***L. aethiopica* 3** |  | 36.4±9.5 | 0.0002 |
| ***L. aethiopica* lab** |  | 33.3±17.4 | 0.0002 |
|  | **Baseline**  **(%)** | **Unassociated**  **(%)** | **^#^p value** |
| ***L. aethiopica* 1** | 7.9±2.2 | 16.9±7.4 | 0.0031 |
| ***L. aethiopica* 2** |  | 13.3±4.8 | 0.0059 |
| ***L. aethiopica* 3** |  | 18.1±3.9 | 0.0002 |
| ***L. aethiopica* lab** |  | 20.1±9.6 | 0.0031 |

1x10^5^ cells/ml neutrophils were cultured in the presence or absence of 1x10^6^ cells/ml FR labelled *L. aethiopica* isolates for 2 hrs. The percentages of apoptotic (as defined by Annexin V^+^7AAD^-^) neutrophils associated or not (baseline) with *L. aethiopica* were determined by flow cytometry. Statistical differences were determined using a Mann-Whitney test (**^#^**)**.**

**Table S2: Comparison of the % of apoptotic neutrophils between unassociated and associated neutrophils following 2hrs of co-incubation with *L. aethiopica***

| **Apoptosis** | **Unassociated**  **(%)** | **Associated**  **(%)** | **^#^p value** |
| --- | --- | --- | --- |
| ***L. aethiopica* 1** | 16.9±7.4 | 32.8±8.8 | 0.0022 |
| ***L. aethiopica* 2** | 13.3±4.8 | 23.0±7.5 | 0.0040 |
| ***L. aethiopica* 3** | 18.1±3.9 | 36.4±9.5 | 0.0003 |
| ***L. aethiopica* lab** | 20.1±9.6 | 33.3±17.4 | 0.1605 |

1x10^5^ cells/ml neutrophils were co-cultured with 1x10^6^ cells/ml FR labelled *L. aethiopica* isolates for 2 hrs. The percentages of apoptotic (as defined by Annexin V^+^7AAD^-^) neutrophils were determined by flow cytometry. Statistical differences were determined using a Mann-Whitney test (**^#^**)**.**

**Table S3: % of apoptotic neutrophils following 18 hrs of incubation**

| **Apoptosis** | **Baseline**  **(% Annexin V^+^ 7AAD^-^ neutrophils)** | **CD15^int^parasites^-^**  **(% Annexin V^+^ 7AAD^-^ neutrophils)** | **^#^p value** |
| --- | --- | --- | --- |
| ***L. aethiopica* 1** | 76±6 | 76±9 | 0.9369 |
| ***L. aethiopica* 2** |  | 74±9 | 0.8974 |
| ***L. aethiopica* 3** |  | 80±3 | 0.1503 |
| ***L. aethiopica* lab** |  | 80±3 | 0.0872 |
|  | **Baseline**  **(% Annexin V^+^ 7AAD^-^ neutrophils)** | **CD15^high^parasites^+^**  **(% Annexin V^+^ 7AAD^-^ neutrophils)** | **^#^p value** |
| ***L. aethiopica* 1** | 76±6 | 57±10 | 0.0017 |
| ***L. aethiopica* 2** |  | 54±12 | 0.0006 |
| ***L. aethiopica* 3** |  | 61±11 | 0.0020 |
| ***L. aethiopica* lab** |  | 43±12 | 0.0002 |
|  | **Baseline**  **(% Annexin V^+^ 7AAD^-^ neutrophils)** | **CD15^low^parasites^+^**  **(% Annexin V^+^ 7AAD^-^ neutrophils)** | **^#^p value** |
| ***L. aethiopica* 1** | 76±6 | 29±16 | 0.0002 |
| ***L. aethiopica* 2** |  | 32±19 | 0.0003 |
| ***L. aethiopica* 3** |  | 40±14 | 0.0002 |
| ***L. aethiopica* lab** |  | 26±13 | 0.0002 |

1x10^5^ cells/ml neutrophils were cultured in the presence or in the absence of 1x10^6^ cells/ml FR labelled *L. aethiopica* isolates for 18 hrs. The percentages of apoptotic (as defined by Annexin V^+^7AAD^-^) neutrophils associated or not (baseline) with *L. aethiopica* were determined by flow cytometry. Statistical differences were determined using a Mann-Whitney test (**^#^**)**.**

**Table S4: Comparison of the % of apoptotic CD15^int^parasite^-^, CD15^high^parasite^+^ and CD15^low^parasite^+^ for each parasites following 18 hrs of incubation**

| ***L. aethiopica* 1** | **% apoptosis** | **^p value** |  | **^#^p value** |
| --- | --- | --- | --- | --- |
| CD15^int^parasites^-^ | 76±9 | 0.0002 | CD15^int^parasites^-^  vs CD15^high^parasites^+^ | 0.1107 |
| CD15^high^parasites^+^ | 57±10 |  | CD15^int^parasites^-^  vs CD15^low^parasites^+^ | 0.0001 |
| CD15^low^parasites^+^ | 29±16 |  | CD15^high^parasites^+^  vs CD15^low^parasites^+^ | 0.1259 |
| ***L. aethiopica* 2** | **% apoptosis** | **^p value** |  | **^#^p value** |
| CD15^int^parasites^-^ | 74±9 | 0.0009 | CD15^int^parasites^-^  vs CD15^high^parasites^+^ | 0.1367 |
| CD15^high^parasites^+^ | 54±12 |  | CD15^int^parasites^-^  vs CD15^low^parasites^+^ | 0.0006 |
| CD15^low^parasites^+^ | 32±19 |  | CD15^high^parasites^+^  vs CD15^low^parasites^+^ | 0.2488 |
| ***L. aethiopica* 3** | **% apoptosis** | **^p value** |  | **^#^p value** |
| CD15^int^parasites^-^ | 80±3 | <0.0001 | CD15^int^parasites^-^  vs CD15^high^parasites^+^ | 0.0360 |
| CD15^high^parasites^+^ | 61±11 |  | CD15^int^parasites^-^  vs CD15^low^parasites^+^ | <0.0001 |
| CD15^low^parasites^+^ | 40±14 |  | CD15^high^parasites^+^  vs CD15^low^parasites^+^ | 0.2307 |
| ***L. aethiopica* lab** | **% apoptosis** | **^p value** |  | **^#^p value** |
| CD15^int^parasites^-^ | 80±3 | 0.0001 | CD15^int^parasites^-^  vs CD15^high^parasites^+^ | 0.0252 |
| CD15^high^parasites^+^ | 43±12 |  | CD15^int^parasites^-^  vs CD15^low^parasites^+^ | <0.0001 |
| CD15^low^parasites^+^ | 26±13 |  | CD15^high^parasites^+^  vs CD15^low^parasites^+^ | 0.3846 |

1x10^5^ cells/ml neutrophils were co-cultured with 1x10^6^ cells/ml FR labelled *L. aethiopica* isolates for 18 hrs. The percentages of apoptotic (as defined by Annexin V^+^7AAD^-^) CD15^int^parasite^-^, CD15^high^parasite^+^ and CD15^low^parasite^+^ were determined by flow cytometry. Statistical differences were determined using Kruskal-Wallis (***)** and Dunn’s multiple comparison (**^**) tests**.**

**Table S5: ROS MFI following 2 hrs of incubation**

| **ROS** | **Baseline**  **(MFI)** | **Associated**  **(MFI)** | **^#^p value** |
| --- | --- | --- | --- |
| ***L. aethiopica* 1** | 324±151 | 898±324 | 0.0043 |
| ***L. aethiopica* 2** |  | 1066±309 | 0.0022 |
| ***L. aethiopica* 3** |  | 1016±367 | 0.0022 |
| ***L. aethiopica* lab** |  | 1674±691 | 0.0022 |
|  | **Baseline**  **(MFI)** | **Unassociated**  **(MFI)** | **^#^p value** |
| ***L. aethiopica* 1** | 324±151 | 411±170 | 0.3896 |
| ***L. aethiopica* 2** |  | 473±153 | 0.0433 |
| ***L. aethiopica* 3** |  | 413±175 | 0.3333 |
| ***L. aethiopica* lab** |  | 398±163 | 0.3312 |

1x10^5^ cells/ml neutrophils were cultured in the presence or absence of 1x10^6^ cells/ml FR labelled *L. aethiopica* isolates for 2 hrs. ROS MFI in neutrophils associated or not (basline) with *L. aethiopica* were determined by flow cytometry. Statistical differences were determined using a Mann-Whitney test (**^#^**)**.**

**Table S6: Comparison of ROS MFI between unassocaited and associated neutrophils following 2hrs of co-incubation with *L. aethiopica***

| **ROS** | **Unassociated**  **(MFI)** | **Associated**  **(MFI)** | **^#^p value** |
| --- | --- | --- | --- |
| ***L. aethiopica* 1** | 411±170 | 898±324 | 0.0173 |
| ***L. aethiopica* 2** | 473±153 | 1066±309 | 0.0043 |
| ***L. aethiopica* 3** | 413±175 | 1016±367 | 0.0043 |
| ***L. aethiopica* lab** | 398±163 | 1674±691 | 0.0022 |

1x10^5^ cells/ml neutrophils were co-cultured with 1x10^6^ cells/ml FR labelled *L. aethiopica* isolates for 2 hrs. ROS MFI were determined by flow cytometry. Statistical differences were determined using a Mann-Whitney test (**^#^**)**.**

**Table S7: ROS MFI in neutrophils following 18 hrs of incubation**

| **ROS** | **Baseline**  **(MFI)** | **CD15^int^parasites^-^**  **(MFI)** | **^#^p value** |
| --- | --- | --- | --- |
| ***L. aethiopica* 1** | 83.33±26.88 | 80.50±25.72 | 0.9740 |
| ***L. aethiopica* 2** |  | 92.83±41.56 | 0.7294 |
| ***L. aethiopica* 3** |  | 98.00±37.34 | 0.4848 |
| ***L. aethiopica* lab** |  | 117.3±43.63 | 0.1580 |
|  | **Baseline**  **(MFI)** | **CD15^high^parasites^+^**  **(MFI)** | **^#^p value** |
| ***L. aethiopica* 1** | 83.33±26.88 | 126.0±45.48 | 0.1017 |
| ***L. aethiopica* 2** |  | 136.7±81.28 | 0.3095 |
| ***L. aethiopica* 3** |  | 159.3±90.29 | 0.0931 |
| ***L. aethiopica* lab** |  | 183.2±83.60 | 0.0649 |
|  | **Baseline**  **(MFI)** | **CD15^low^parasites^+^**  **(MFI)** | **^#^p value** |
| ***L. aethiopica* 1** | 83.33±26.88 | 40.00±12.43 | 0.0087 |
| ***L. aethiopica* 2** |  | 30.83±8.448 | 0.0022 |
| ***L. aethiopica* 3** |  | 56.17±16.18 | 0.0455 |
| ***L. aethiopica* lab** |  | 65.33±17.05 | 0.3701 |

1x10^5^ cells/ml neutrophils were cultured in the presence or in the absence of 1x10^6^ cells/ml FR labelled *L. aethiopica* isolates for 18 hrs. ROS MFI in neutrophils associated or not (baseline) with *L. aethiopica* were determined by flow cytometry. Statistical differences were determined using a Mann-Whitney test (**^#^**)**.**

**Table S8: Comparison of ROS MFI in CD15^int^parasite^-^, CD15^high^parasite^+^ and CD15^low^parasite^+^ for each parasites following 18 hrs of incubation**

| ***L. aethiopica* 1** | **ROS (MFI)** | **^p value** |  | **^#^p value** |
| --- | --- | --- | --- | --- |
| CD15^int^parasites^-^ | 81±26 | 0.0003 | CD15^int^parasites^-^  vs CD15^high^parasites^+^ | 0.5543 |
| CD15^high^parasites^+^ | 126±45 |  | CD15^int^parasites^-^  vs CD15^low^parasites^+^ | 0.1191 |
| CD15^low^parasites^+^ | 40±12 |  | CD15^high^parasites^+^  vs CD15^low^parasites^+^ | 0.0022 |
| ***L. aethiopica* 2** | **ROS (MFI)** | **^p value** |  | **^#^p value** |
| CD15^int^parasites^-^ | 93±42 | 0.0003 | CD15^int^parasites^-^  vs CD15^high^parasites^+^ | >0.9999 |
| CD15^high^parasites^+^ | 137±81 |  | CD15^int^parasites^-^  vs CD15^low^parasites^+^ | 0.0260 |
| CD15^low^parasites^+^ | 31±8 |  | CD15^high^parasites^+^  vs CD15^low^parasites^+^ | 0.0038 |
| ***L. aethiopica* 3** | **ROS (MFI)** | **^p value** |  | **^#^p value** |
| CD15^int^parasites^-^ | 98±37 | **^**0.0099 | CD15^int^parasites^-^  vs CD15^high^parasites^+^ | 0.5293 |
| CD15^high^parasites^+^ | 159±90 |  | CD15^int^parasites^-^  vs CD15^low^parasites^+^ | 0.3900 |
| CD15^low^parasites^+^ | 56±16 |  | CD15^high^parasites^+^  vs CD15^low^parasites^+^ | 0.0125 |
| ***L. aethiopica* lab** | **ROS (MFI)** | **^p value** |  | **^#^p value** |
| CD15^int^parasites^-^ | 117±44 | 0.0130 | CD15^int^parasites^-^  vs CD15^high^parasites^+^ | 0.7004 |
| CD15^high^parasites^+^ | 183±84 |  | CD15^int^parasites^-^  vs CD15^low^parasites^+^ | 0.3304 |
| CD15^low^parasites^+^ | 65±17 |  | CD15^high^parasites^+^  vs CD15^low^parasites^+^ | 0.0159 |

1x10^5^ cells/ml neutrophils were co-cultured with 1x10^6^ cells/ml FR labelled *L. aethiopica* isolates for 18 hrs. ROS MFI in CD15^int^parasite^-^, CD15^high^parasite^+^ and CD15^low^parasite^+^ were determined by flow cytometry. Statistical differences were determined using Kruskal-Wallis (***)** and Dunn’s multiple comparison (**^**) tests**.**
