# Supplementary figures and images for: Distinct neutrophil effector functions in response to different isolates of Leishmania aethiopica
Source: Parasit Vectors. 2024 Nov 11;17:461. doi: 10.1186/s13071-024-06489-x (PMC11555981; doi:10.1186/s13071-024-06489-x)

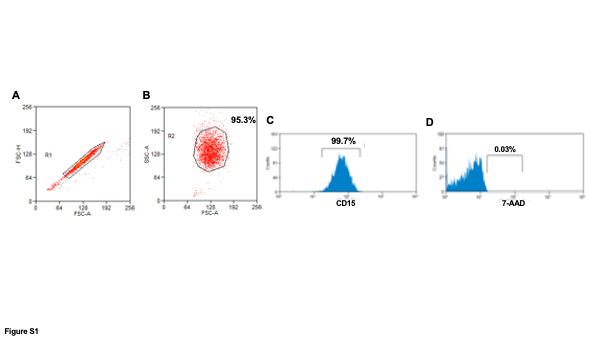

Supplement: Supplementary file 2 — Supplementary Material 2. [file 13071_2024_6489_MOESM2_ESM.tiff]

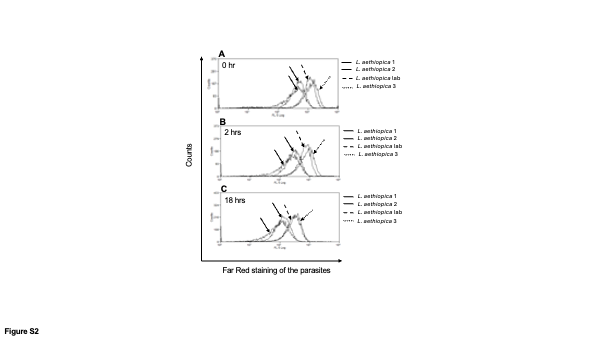

Supplement: Supplementary file 3 — Supplementary Material 3. [file 13071_2024_6489_MOESM3_ESM.tiff]

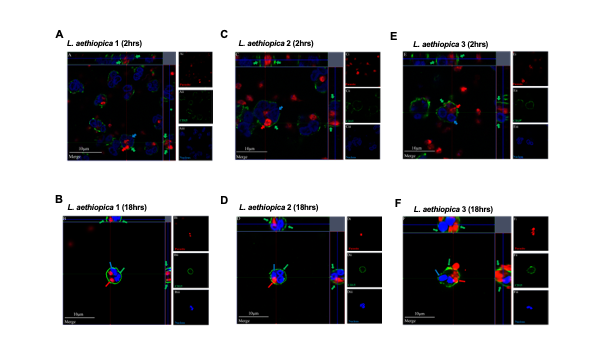

Supplement: Supplementary file 4 — Supplementary Material 4. [file 13071_2024_6489_MOESM4_ESM.tiff]

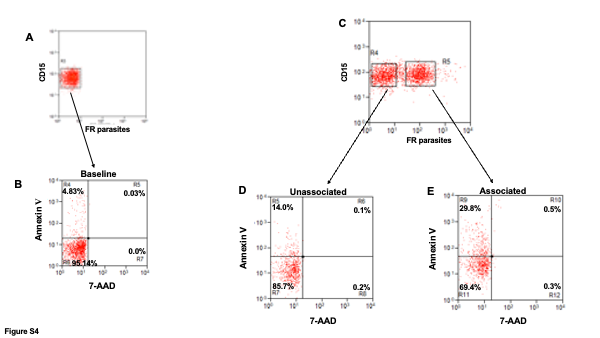

Supplement: Supplementary file 5 — Supplementary Material 5. [file 13071_2024_6489_MOESM5_ESM.tiff]

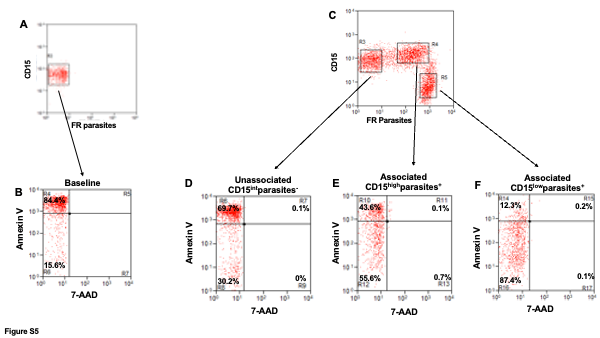

Supplement: Supplementary file 6 — Supplementary Material 6. [file 13071_2024_6489_MOESM6_ESM.tiff]

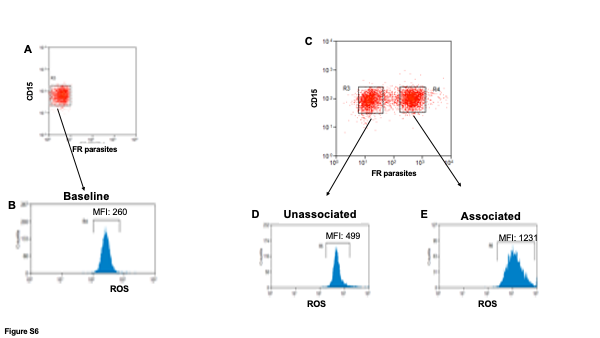

Supplement: Supplementary file 7 — Supplementary Material 7. [file 13071_2024_6489_MOESM7_ESM.tiff]

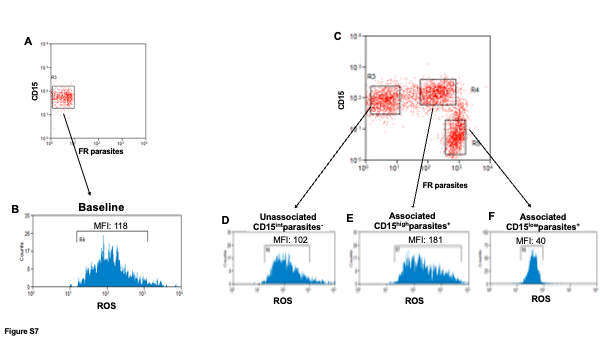

Supplement: Supplementary file 8 — Supplementary Material 8. [file 13071_2024_6489_MOESM8_ESM.tiff]
